# A Comprehensive Mechanical and Chemoprophylaxis Algorithm for Prevention of Venous Thromboembolism in Lipoabdominoplasty

**DOI:** 10.1093/asjof/ojaf024

**Published:** 2025-04-16

**Authors:** R Brannon Claytor, Grace Tolan, Trevor Pettibone, Alec Fisher

## Abstract

**Background:**

Because the desire for body contouring rises exponentially, the rate of abdominoplasty continues to increase. Although this procedure provides patients with aesthetically pleasing results, pulmonary embolism (PE) represents a potentially fatal risk surgeons seek to avoid with risk stratification and prophylaxis based on the 2005 Caprini risk assessment model (RAM). Despite the efforts of the American Society of Plastic Surgery task force, much uncertainty exists on the appropriate venous thromboembolism (VTE) prophylaxis.

**Objectives:**

The aim of this study is to demonstrate the safety and efficacy of utilizing a comprehensive mechanical and chemoprophylaxis protocol to prevent VTE in abdominoplasty.

**Methods:**

This was a retrospective study reviewing 1 surgeon's (R.B.C.) postoperative complications for 333 patients who underwent abdominoplasty, belt lipectomy, or modified float tummy tuck from January 2017 to April 2024. All patients received chemoprophylaxis consisting of preoperative heparin injection, intraoperative intermittent pneumatic compression (IPC) devices, 1 week of postoperative enoxaparin injections and home IPCs for 2 weeks. All patients were preoperatively screened using the 2005 Caprini RAM; high-risk patients (≥6) continued enoxaparin injections for 1 month postoperatively.

**Results:**

The median 2005 Caprini score was 4. There were 34 (10.2%) complications postoperatively: 2 (0.60%) PEs, 5 (1.50%) seromas, 6 (1.80%) hematomas, 3 (0.90%) wound healing complications, and 12 (3.60%) wound infections. The PE events occurred in patients with a 2005 Caprini score of 4.

**Conclusions:**

The 2005 Caprini RAM provides a framework to guide VTE prophylaxis; however, patients with low/moderate-risk scores may suffer deep vein thrombosis (DVT) or PE. The authors demonstrate that a comprehensive mechanical and chemoprophylaxis protocol reduced the incidence of VTE compared with the literature and did not increase the risk of bleeding or complication.

**Level of Evidence: 3 (Therapeutic):**

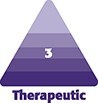

Pulmonary embolism (PE) reigns as the nation's leading cause of preventable hospital death and is a catastrophic complication of abdominoplasty that must not be overlooked.^1^ Improvements in diet, exercise, and pharmacology have contributed to increased rates of successful weight loss journeys, leaving excess abdominal skin and laxity. Abdominoplasty is the definitive procedure to provide patients with their desired abdominal contour and has never been more sought after, ranking as the third most popular cosmetic procedure in 2023.^2^ A consequential relationship between abdominoplasty and venous thromboembolism (VTE) has resulted in both considerable and appropriate attention in the literature, with publications reporting rates as high as 2.17%.^3,4^ The exact etiology of the heightened risk of PE in abdominoplasty patients remains elusive, although experts have speculated factors including, but not limited to, the duration of the operation, high BMI, angle of flexion on operating bed, postoperative abdominal binders, surgical techniques, rectus plication, and hematologic genetic mutations.^5-16^

As the etiology of PE remains complicated, so does the utilization of the proper risk assessment model (RAM) and subsequent prophylaxis regimen. Although much research and discussion have been done regarding proper VTE prophylaxis for the plastic surgery population, no universal consensus has been reached, and no rigid guidelines have been published.^7,17-23^ The current accepted model is the 2005 Caprini RAM, stratifying patients as high or low/moderate risk based on self-reported risk factors and health history.^24^ The RAM suggests guidelines for mechanical and chemoprophylaxis for high-risk patients. Even with extensive evaluation and subsequent recommendations added to the 2005 Caprini RAM by the American Society of Plastic Surgery task force in 2011, a standardized chemoprophylaxis regimen for abdominoplasty patients remains ambiguous.^17^ Some surgeons avoid chemoprophylaxis, relying solely on mechanical prophylaxis (intermittent pneumatic compression [IPCs]), early ambulation, and other low-bleeding-risk approaches out of concern for postoperative bleeding and hematomas.^7,25,26^ Despite this, many surgeons have found the implementation of postoperative anticoagulation to be effective in reducing the incidence of fatal PE, and combinations of mechanical or chemoprophylaxis deliver even more comprehensive prophylaxis.^27-33^ A reasonable strategy is to avoid operating on “high-risk” patients, and therefore, ∼97% of patients are stratified as low/moderate risk by the 2005 Caprini RAM.^7,17,34^ The data, however, show that 67% of VTEs have occurred in low/moderate-risk patients.^18,35,36^ Thus, it is imperative for public health safety that investigation into protecting the low/moderate-risk population be evaluated. The purpose of this study is to investigate the risks and benefits of utilizing a comprehensive preoperative and postoperative mechanical and chemoprophylaxis regimen that is standardized for all abdominoplasty patients regardless of 2005 Caprini risk assessment score with the intent to eliminate fatal PEs while simultaneously not increasing the incidence of postoperative hematomas or bleeding complications.

## METHODS

This retrospective study was approved by the Main Line Health Institutional Review Board E-24-5409. Patients were identified as individuals who had undergone abdominoplasty, belt lipectomy, or modified float abdominoplasty performed by the senior surgeon (R.B.C.) between January 2017 and April 2024. All operations were performed in the hospital setting as outpatient procedures, and patients were discharged the same day. All procedures involved plication of the rectus muscles with or without concomitant procedures. Outcome metrics analyzed included procedure time, lipoaspirate volume, weight of resected tissue, and complications. Mean and standard deviation were analyzed through Microsoft Excel data sheets (Microsoft Corporation, Redmond, WA). Chi-squared analysis, Mann–Whitney *U* analysis, and analysis for statistical significance were performed through IBM-SPSS 29.0 software (IBM Corporation, Armonk, NY).

**Figure 1.**
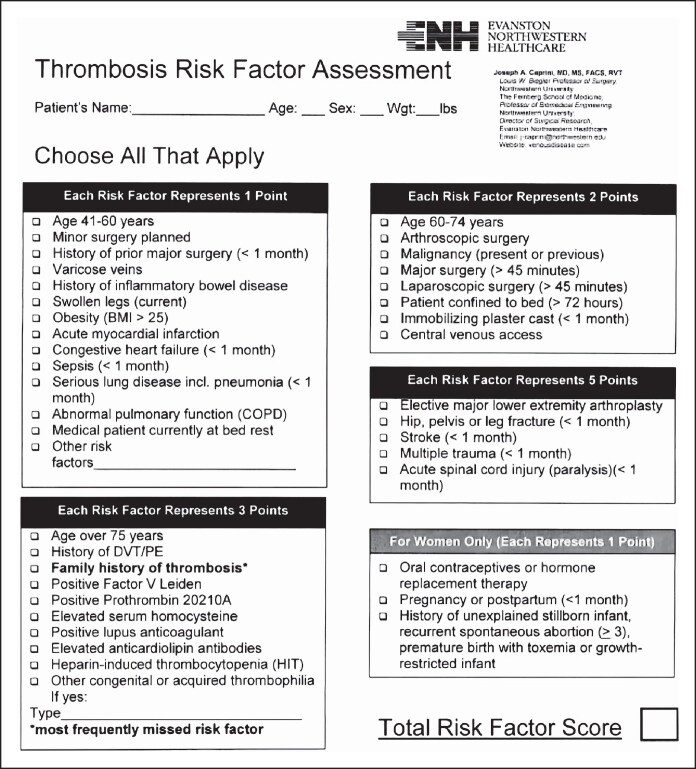
2005 Caprini risk assessment model. Reproduced with permission by Elsevier from Caprini.^34^

All patients were screened through the 2005 Caprini RAM (Figure 1). If a patient had a personal history of blood clots, hematologic disorders, or a family history of a first-degree relative with a history of blood clots, a preoperative hematology evaluation was mandatory. This was done by a hematologist and included a genetic workup for Factor V Leiden, Proteins C and S deficiency, antithrombin III, aPTT, anticardiolipin antibody, prothrombin gene mutation, Factor VII activity, and homocysteine. Patient demographics including age, gender, BMI, 2005 Caprini RAM score, concomitant procedures, and personal and/or family history of hematologic disease were recorded.

All abdominoplasties were performed by the senior surgeon in the same fashion: a solution of tumescent fluid consisting of 30 cc 1% lidocaine and 1:1000 (1 mg/mL) epinephrine per liter was injected. Specific surgical techniques included liposuction to the abdomen and flanks, preservation of Scarpa's fascia, elevation of the abdominal flap to the xiphoid. Elevation of the abdominal flap is carried out by sharp dissection with a #10 blade scalpel and minimal usage of electrocautery to achieve hemostasis of identifiable blood vessels. Plication of the rectus muscles with interrupted 0 prolene sutures, running 2-0 prolene, and a final layer of running 2-0 polydioxanone (PDS). Progressive tension sutures to close off any open space were placed with interrupted 2-0 nylon. Closure was performed without surgical drains. At the conclusion of the operation, the patient is placed in an abdominal binder, which is tightened and marked to a moderately tight amount of compression.

All patients were treated with the same mechanical and chemoprophylaxis regimen. The studied protocol is composed of a preoperative 5000-unit heparin injection ∼1 h before their operation and once-a-day postoperative 40 mg enoxaparin injections for 1 week for low/moderate-risk patients (2005 Caprini <6). Anticoagulation is held until 24 h postoperatively. At 24 h postoperative, the patient is instructed to upload photographs to TouchMD (TouchMD, Cedar City, UT) to evaluate for ecchymosis. If there is demonstratable ecchymosis, enoxaparin is held for an additional 24 h and then initiated at postoperative Day 2. Patients are asked to return for postoperative clinic visits every week for the first month postop, then every month for the next 6 months. After patients are 6 months postoperative, they are advised to follow-up every 6 months. No tissue adhesive or tranexamic acid (TXA) was used in this cohort. In high-risk patients (2005 Caprini ≥6), enoxaparin injections are continued for 1 month. IPC devices are worn for ∼1 h in the preoperative waiting room, intraoperatively, and for at least 2 weeks postoperatively. The patients are expected to wear the devices 24/7 and compliance is monitored through patient reports at postoperative visits. Incentive spirometers are provided at no cost, and patients are instructed to use them 10 times/h for 1 week postoperatively.

## RESULTS

**Figure 2.**
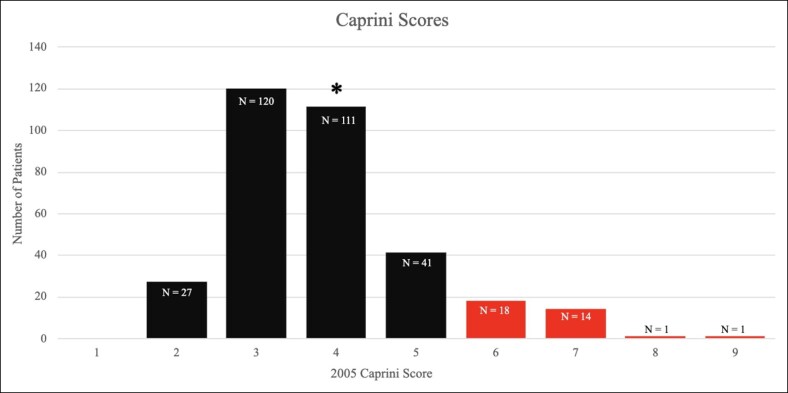
Histogram of patient's preoperative 2005 Caprini risk assessment model scores. Columns 6-9 represent the patients who were considered high risk (≥6). *2005 Caprini scores of the 2 patients who developed postoperative PEs.

Three hundred and thirty-three patients over a 7-year period were retrospectively reviewed, and the demographic and surgical data are outlined in Table 1. The study cohort had an average age of 46 years (range, 24-78 years) and an average BMI of 27.7 (range, 18.2-43.3). The median 2005 Caprini score was 4 (range, 2-9; Figure 2). Thirty-four (10.2%) patients of 333 were considered high risk based on the 2005 Caprini score (≥6). The entire cohort was then divided into low/moderate-risk (2-5) and high-risk (≥6) cohorts, and the data were compared to determine statistical significance using a *P*-value of <.05 (Table 1). No characteristics were found to differ significantly between the low/moderate-risk and high-risk cohorts. Twenty-five (7.51%) patients were required to see a hematologist before surgery because of either a personal history of clotting disorder, previous VTE, or a history of a first-degree relative who suffered a VTE. The hematology consult was done to provide further recommendations on prophylaxis and clearance for surgery. All patients who underwent preoperative consultation were cleared for surgery, and no operations were delayed or canceled based on this evaluation.

**Table 1.** General Characteristics of Study Group

	All patients (*n* = 333)	Low/moderate risk (2005 Caprini 2-5) (*n* = 299)	High risk (2005 Caprini ≥6) (*n* = 34)	*P*-value (<.05)
Age, years				.189
Mean ± SD	46 ± 10.06	45.47 ± 9.807	47.85 ± 11.781	
Range	24-78	24-78	28-76	
BMI, kg/m^2^				.159
Mean ± SD	27.72 ± 4.85	27.59 ± 4.94	28.83 ± 3.83	
Range	18.20-43.30	18.20-43.30	22.60-34.86	
Weight, kg				.382
Mean ± SD	74.7 ± 15.3	74.5 ± 15.5	76.9 ± 13.9	
Range	45.8-136.1	45.8-136.1	55.8-113.4	
Average procedure time (min)				.879
Mean ± SD	258.7 ± 69.28	258.14 ± 69.08	260.06 ± 69.93	
Range	120-485	120-485	149-383	
Weight of lipocutaneous flap (g)				.131
Mean ± SD	2103.8 ± 1364.2	2064.5 ± 1349.8	2462.3 ± 1461.1	
Range	130-12,700	130-12,700	350-7257	
Lipoaspirate volume (cc)				.462
Mean ± SD	1123.5 ± 724.5	1115.72 ± 701.3	1215.6 ± 899.5	
Range	20-4000	20-4000	200-4000	

A Student's *t* test was used to determine significance between the low/moderate-risk cohort and the high-risk cohort. A *P*-value of <.05 was used to determine statistical significance. SD, standard deviation.

Two hundred and twenty-three patients (67.0%) underwent concomitant procedures, most commonly bilateral mastopexy (see Supplemental Table 1). The average operative time was 258.7 min (range, 120-485 min). Forty-three (12.9%) patients reported current use of oral or systemic hormones and were instructed to stop usage 2 weeks before surgery. Thirteen (3.9%) patients identified as current smokers and were instructed to quit 2 weeks before their operation. Patients were stratified into high- and low/moderate-risk groups, and a Mann–Whitney *U* analysis was performed and found a statistically significant difference in BMI (*P* = .048). No other risk factor included in the analysis was found to differ significantly between groups (Table 2).

**Table 2.** Mann–Whitney *U* Test to Determine Significance of Venous Thromboembolism Risk Factors Between Low- and High-Risk (≥6) Patients Based on 2005 Caprini Risk Assessment Model

VTE risk factor	Low/moderate risk (*n* = 299) (mean ± SD)	High risk (*n* = 34) (mean ± SD)	*P*-value (<.05)
Age	45.43 ± 9.80	48.09 ± 11.88	.352
Procedure length (min)	258.22 ± 69.25	262.48 ± 69.54	.997
Body weight (kg)	74.5 ± 15.5	76.9 ± 13.9	.258
BMI	27.64 ± 4.94	28.75 ± 3.86	.048^a^
Lipoaspirate volume (cc)	1115.98 ± 700.68	1229.03 ± 911.11	.751
Weight of lipocutaneous flap (g)	2073.29 ± 1350.91	2443.72 ± 1483.32	.171

SD, standard deviation; VTE, venous thromboembolism. ^a^*P-*value of <.05 was used to determine statistical significance.

Thirty-four (10.2%) patients had complications following surgery, and there were zero mortalities (Table 3). Twelve (3.6%) patients developed wound infections and were treated conservatively with appropriate antibiotics, resulting in complete resolution. Five (1.5%) patients experienced postoperative seroma, 4 of which were aspirated in the clinic and 1 requiring return to the operating room (OR) for drain placement. All patients recovered with no additional sequelae. Six (1.8%) patients developed hematomas: 5 were drained in clinic and 1 required incision and drainage in the OR with placement of a negative pressure vacuum-assisted closure device (KCI Technologies, Sparks, MD). Following appropriate treatment, all patients recovered with optimal results.

**Table 3.** A Table of Postoperative Complications

	All patients (*n* = 333), *n* (%)
Overall complications	34 (10.2)
Infection	12 (3.6)
Hematoma	6 (1.8)
Seroma	5 (1.5)
Pneumonia	4 (1.2)
Wound healing complications	3 (0.9)
PE	2 (0.6)
Ileus	2 (0.6)

Infection was included if the affected site was confirmed with cultures. Seroma was defined as any fluid collection >25 cc. Wound healing complications included skin necrosis and open wounds. PE, pulmonary embolism.

Eight (2.4%) patients reported shortness of breath following surgery and were sent to the emergency department to rule out PE. On average, patients reported developing symptoms on postoperative Day 3.6 (range, 1-13 days). Of these patients, 2 patients were positive for PE, 4 were found to have pneumonia, and 2 had ileus. It should be noted that early pulmonary symptoms are common for patients undergoing surgery, and on radiograph, atelectasis can sometimes be incorrectly read as pneumonia.

Two (0.6%) patients suffered from a PE postoperatively. Patient 1 had abdominoplasty alone, and Patient 2 had abdominoplasty with 360 liposuction and fat grafting to the bilateral breast. Patient 1 had an operative time of 240 min and Patient 2 had 270 min. Both patients had 2005 Caprini score of 4, and thus were low/moderate risk. Patient 1's clinical course is listed as follows: on postoperative Day 5, she developed tachypnea and had an episode of syncope. She was transported to the emergency room by ambulance and spiral computed tomography (CT) scan diagnosed acute PE with cor pulmonale. Additionally, she was found to have a mobile, nonocclusive DVT in the left common femoral vein. The patient was treated with an endovascular thrombectomy of an 8-inch saddle embolus and was placed on long-term oral anticoagulation. Patient 2 was symptomatic with dyspnea and diagnosed by spiral CT with a PE on postoperative Day 25. The therapeutic intervention required was intravenous heparin with a bridge to oral anticoagulation. Following recovery, both patients underwent hematology evaluation; neither was found to have any genetic mutations or coagulopathies. Fortunately, both patients recovered without long-term sequela. Of note, after the fact, Patient 2's mother shared a history of PE. Had this been identified preoperatively, the patient would have had a 2005 Caprini of 7 and undergone a preoperative hematology workup and continued enoxaparin injections for 1 month after surgery.

## DISCUSSION

PE is the primary cause of mortality associated with VTE and often results in sudden death.^1,32,37^ The interest in achieving the ideal balance between VTE prophylaxis and avoidance of bleeding complications is our ever-present goal. Because no standardized protocol for prophylaxis exists, the potential for a fatal PE remains high and is a critical concern.^38^ It is imperative to patient safety that the efficacy of a comprehensive prophylaxis regimen be further investigated. This study demonstrates the utilization of a comprehensive preoperative and postoperative mechanical and chemoprophylaxis regimen can be safely implemented without increasing the risk of bleeding events such as hematoma.

The rationale behind the studied prophylaxis regimen is based on a culmination of evidence-based practices described in the literature. The decision to implement anticoagulation before surgery with a 5000-unit heparin injection is based on the data published in the Campbell et al study, along with acknowledgment that blood clot formation begins at the induction of anesthesia.^31,39^ The inclusion of perioperative IPC devices is founded on basic tenets of well-established DVT prophylaxis, which represents the universally accepted standard of care.^40^ The use of IPCs for 2 weeks postoperatively is a continuation of perioperative prophylaxis and in recognition that the ongoing potential risk for VTE is highest in the postoperative period.^39^ A low-tech passive VTE prophylaxis ensures comprehensive protection during the most vulnerable period of time. The initiation of postoperative enoxaparin is again well described in the literature and has been used extensively for VTE prophylaxis. Anticoagulation is begun 24 h postoperatively to minimize the risk of bleeding complications. Given that these patients received preoperative heparin injections, the delayed initiation of enoxaparin can be justified as a part of a combined treatment approach. We elect to use subcutaneous enoxaparin injections as opposed to direct oral anticoagulants as this has been the historic recommendation, and oral anticoagulants are expensive and not as easily reversed when managing postoperative bleeding complications.^39^ Our protocol mandates 1 week of postoperative enoxaparin for all patients, irrespective of their 2005 Caprini RAM score. Our approach acknowledges that the 2005 Caprini score may not adequately identify higher risk patients, and it suggests a uniform treatment algorithm for universal prophylaxis. The decision to extend postoperative anticoagulation for 1 month in patients with a 2005 Caprini score of ≥6 is driven by the understanding that these patients have risk factors that elevate their risk beyond that of the average patient, thereby necessitating a longer treatment algorithm. Classifying medical histories like congestive heart failure or an acute myocardial infarction with the same level of severity as a varicose vein is missing a significant understanding of what may contribute to the incidence of VTE. Therefore, we chose to define a 2005 Caprini score ≥6 as high risk, despite the existing literature, to ensure comprehensive coverage for any eventuality. The integration of these measures highlights that each one provides a significant contribution to risk reduction. Our study demonstrates that, despite breakthrough PEs under this protocol, there were no mortalities.

Studies have found that the implementation of preoperative and/or postoperative chemoprophylaxis does not have a statistically significant impact on the incidence of VTE.^41^ However, in a systematic review of 1596 patients, there was still a 0.56% incidence of VTE.^41^ Although not statistically significant in numbers, this is still 9 patients who experienced a VTE event, which may or may not have resulted in mortality. The question the authors are attempting to address regarding the appropriateness of the 2005 Caprini score for risk stratification is whether or not all patients can be adequately protected from mortality secondary to VTE regardless of their risk factors. Best said by Dr Pannucci, “unlike complex and comorbid plastic surgery inpatients, the elective aesthetic population is typically younger and healthier. Although VTE is rare among these individuals, a fatal pulmonary embolus in a 35-year-old mother of three is devastating in multiple paradigms.”^40^ Perhaps a less aggressive protocol can prevent VTE just as well as the proposed method; however, this study suggests implementation of far more aggressive chemo- and mechanical prophylaxis protocols than the current accepted regimens is a safe and effective measure in preventing VTE. In doing so, surgeons may be able to provide widespread protection against mortality secondary to PE while having minimal, if any, impact on hematoma incidence. However, the full implications of the proposed mechanical and chemoprophylaxis regimen can only be definitively understood once controlled, multicentered studies are performed.

The 2005 Caprini RAM has been largely critiqued in the literature, and no consensus on a standardized algorithm to prevent PE has been reported.^18,23,35,42^ Currently, the 2005 Caprini RAM is the gold standard for prophylaxis management. As is evident in our study and the published research, a vast majority of patients are stratified as low/moderate risk, and yet paradoxically, this cohort has a higher numerical incidence of PE than high-risk patients.^18,35,36^ Critics may attribute this to low/moderate-risk patients comprising ∼90% to 97% of the abdominoplasty patient population; however, this is exactly the point this study aims to illuminate. Dr Pannucci famously said, “VTE prophylaxis in surgical patients currently falls under the ‘one size fits all’ approach that fails to consider patient-level variation in the risks and benefits of an intervention.”^43^ Although most patients are stratified as low/moderate risk, there is a small majority of unknowingly high-risk patients camouflaged within the low/moderate-risk cohort and therefore would require a more extensive prophylaxis regimen.^40^

The literature has proven plication of the abdominal wall increases intraabdominal pressure and has been shown to increase lower extremity venous stasis.^44^ Therefore, because most surgeons include rectus plication in their abdominoplasties, the procedure alone puts patients at higher risk of VTE development. It is important to note the 2005 Caprini RAM does not consider rectus plication in risk stratification. A novel concept to improve the effectiveness of the 2005 Caprini score suggested by Restifo would add 2 points to the score if rectus plication is part of the abdominoplasty procedure.^7^ Because an integral element of abdominal contouring with abdominoplasty involves the repair of the rectus diastasis, Restifo's suggestion may advance more patients into the high-risk category and help clarify the obscure prophylaxis recommendations. Therefore, more patients would be treated with a more comprehensive prophylaxis protocol.^17^

The rationale for our comprehensive mechanical and chemoprophylaxis protocol is in acknowledgment that fatalities have occurred following abdominoplasties. There is no distinct consensus across our specialty or even recommendations from our societies as to clear-cut protocols that include all 2005 Caprini score patients. We believe the guidelines should place emphasis on preventing PE-induced mortality while at the same time demonstrating minimal postprocedure hematomas; however, the current accepted model is inadequate in this regard. Our protocol acknowledges that despite the utilization of algorithms promoted by RAMs for reduced PEs and mortalities, current protocols do not provide a comprehensive plan to address the possibility of low/moderate-risk patients with unidentifiable risk factors.

In our cohort, Patient 1 experienced a symptomatic, life-threatening saddle PE on postoperative Day 5 while on prophylactic anticoagulation. Some of the shortcomings of the 2005 Caprini score are laid bare here by her statistics. Patient 1 had a 2005 Caprini score of 4 because her BMI was 25.3 and age was 45. These 2 factors are marginally above the cutoff of a BMI of 25 and an age of 41 and were much closer to a 2005 Caprini score of 2. In addition, her hypercoagulable workup was negative for any genetic mutations. This does pose skepticism to the efficacy of our comprehensive prophylaxis regimen; however, in any reasonable algorithm, she would have been considered the lowest of all risk categories, and no mechanical or chemoprophylaxis would have been considered appropriate, and yet she came very close to a fatal condition despite comprehensive prophylaxis. This treatment may very well have saved her life. If a patient with a score of nearly 2 comes close to a fatal PE, while on enoxaparin, it is unclear what protective prognostic utility the 2005 Caprini score has.

**Figure 3.**
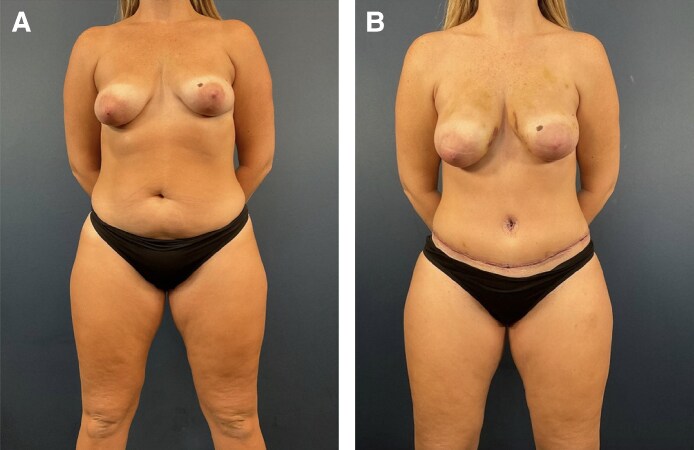
Thirty-six-year-old patient status postabdominoplasty with rectus plication and bilateral flank liposuction and fat grafting to the bilateral breast (A) preoperatively and (B) 2 weeks postoperatively. The patient's preoperative BMI was 26.3. This patient had a 2005 Caprini score of 4, and her prophylaxis regimen included a 5000-unit preoperative heparin injection and 1 week of Lovenox postoperative. Intermittent compression devices were worn preoperatively, intraoperatively, and 2 weeks postoperatively. This patient developed a pulmonary embolism Day 25 postop (Patient 2).

**Figure 4.**
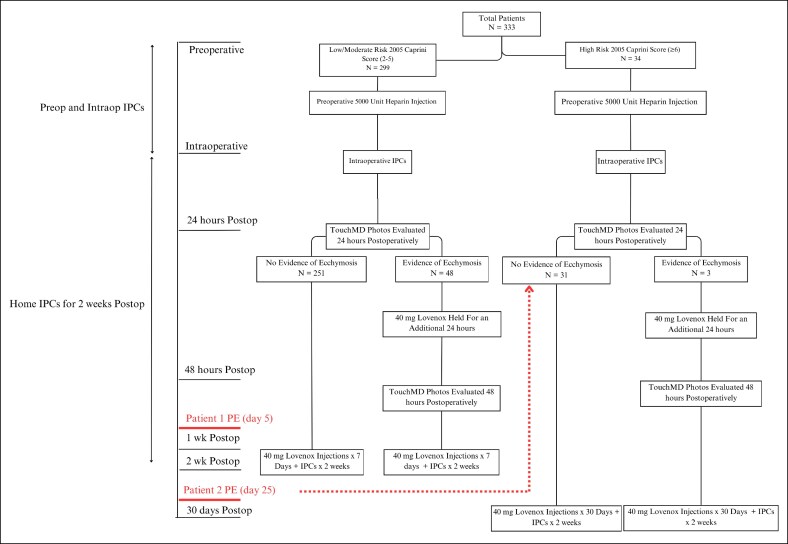
The senior surgeon's mechanical and chemoprophylaxis regimen for abdominoplasty based on their 2005 Caprini risk assessment model score with associated timeline. The dotted arrow represents the group Patient 2 would have been stratified in had she known of her family history of venous thromboembolism.

To further make this point, Patient 2, who had similar circumstances leading to a 2005 Caprini score of 4, reveals the shortcomings personal and family history have when it comes to risk analysis. Had Patient 2 known her mother had a PE, this would have dramatically changed her risk profile and, in our algorithm, would have prompted a hypercoagulable workup as well as 1-month postoperative enoxaparin (Figure 3). In this scenario, we hypothesize it is probable she might have had an earlier or even more severe PE had she not been on enoxaparin for 1 week postoperatively. Knowledge of her family history would have increased her Caprini score to a 7, and she would have been on enoxaparin for 1 month, and therefore, we speculate she might not have developed a PE at all (Figure 4).

It is not necessary to make sweeping proclamations and judgments about treatment algorithms based on the results of 1 patient, it does allow for speculation that our patient, who had a massive saddle embolus on postoperative Day 5 at least had mechanical and chemical thromboembolism prophylaxis actively on board during her potentially life-threatening event. Although it cannot be determined with certainty that these maneuvers were singularly responsible for averting a mortality, it can undoubtedly be said that active mechanical prophylaxis and enoxaparin chemoprophylaxis represent a known and agreed upon preventative DVT measure.

It should also be mentioned that 15% to 25% of untreated DVTs will propagate proximally, increasing the risk of fatal PE.^45-48^ In our study, 1 patient (0.33%) was found to have a DVT that progressed to a life-threatening saddle PE on postoperative Day 5. Some surgeons have utilized Doppler ultrasound as a method to monitor for deep vein thrombosis development postoperatively. Their results show a rate of 0.5% asymptomatic DVTs detected on average at postoperative Day 4.8.^49^ It is our belief that our chemoprophylaxis protocol greatly contributes to the prevention of DVT development because our patients are placed on anticoagulation for at least 1 week postoperatively. We hypothesize that it is because of our comprehensive preoperative and postoperative prophylaxis protocol that we had no symptomatic DVTs in 333 patients, except in the 1 patient who had a near-fatal pulmonary embolus.

**Figure 5.**
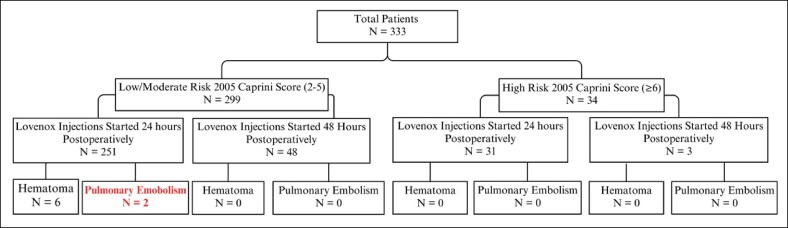
Diagram of postoperative hematoma and pulmonary embolism events based on 2005 Caprini risk assessment model stratification.

The counter argument to such comprehensive prophylaxis is the assumption that chemoprophylaxis will increase the rate of postoperative hematomas or even the concern of a life-threatening postoperative bleed. In some publications, the authors suggest hematoma rates ranging from 0.6% to 3% with some going as high as 12.8%.^22,38,42,50,51^ Although this is a valid concern, our hematoma rate of 1.8% does not demonstrate an increased incidence. In our cohort, only one patient (0.33%) who developed a hematoma required return to the OR and recovered with no long-term sequalae. All other hematomas were managed conservatively (Figure 5). With effective intraoperative hemostasis and comprehensive local anesthetic for pain control, a hematoma is a minor and manageable complication.^14^ Best stated by Davison and Massoumi, “a hematoma is a medical stress, an inconvenience, an embarrassment, or an additional procedure, but rarely does it kill a patient. Thromboembolism that progresses to a pulmonary embolism kills the patient 50 percent of the time.”^52^

Alongside alterations in chemoprophylaxis protocols, additional techniques have been optimized to mitigate the risk of minor postoperative hematomas. These techniques include, but are not limited to, utilizing sharp dissection as opposed to electrocautery, preservation of Scarpa's fascia, progressive tension sutures, and practicing meticulous hemostasis before closure.^11^ However, because the etiology of VTE is ambiguous, even with the implementation of these techniques, the risk of PE still exists. Additionally, tissue adhesives and/or TXA are utilized to prevent hematoma formation. It should be noted the senior surgeon did not use any exogenous coagulators because their contribution to thromboembolism formation remains undetermined.

The 2005 Caprini RAM may adequately identify patients who are at higher risk for developing a VTE; however, its limitations for risk assessment in the low/moderate-risk population are well described, and unrecognized predictors of a potentially fatal PE are still a concern. Although RAMs can be helpful in risk reduction management, they cannot prevent all complications. The 2005 Caprini scale is no exception. We do not advocate for the discontinuation of the 2005 Caprini RAM nor do we advocate for the modification of the point system, but we do acknowledge the shortcomings for low-risk individuals who represent the highest incidence of PE patients. Our recommendation is for the establishment of treatment algorithms that are more comprehensive and demonstrate minimal complications.

There are several limitations in our study worth considering. This was a retrospective study with a small patient cohort comprised of 96.7% female patients. In our study, there was no control group for outcome comparison and this was a single surgeon, single-center study. The suggested conclusions made as a result of this study need to be validated with controlled, multicenter studies. Our report presents results from a single practice using 1 surgeon's techniques and does not imply that the proposed regimen will be effective for everyone, until further controlled studies can be performed to confirm or refute its efficacy.

There were several other factors that could contribute to the etiology of PE that were not analyzed in this study. First, this was an outpatient hospital cohort for an ambulatory surgical procedure, thereby limiting patient ambulation. The British National Formulary suggests hormones be stopped 4 to 6 weeks before major surgery or when long postoperative immobilization is anticipated.^53^ This recommendation is still under speculation, and our patients are ambulatory before discharge; thus, hormones are only held 2 weeks before surgery, which could have impacted the study outcomes. Postoperative enoxaparin injections were held an additional 24 h in 15.3% of patients included in the study based on evidence of ecchymosis at postoperative Day 1; however, none of these patients developed hematomas. Intraoperative anesthesia was not reviewed, but generally, the senior surgeon requested no paralytics during surgery which could contribute to the decreased incidence of VTE. Published studies suggest that fewer paralytics translate to a lower incidence of VTE.^15,54^ Intraabdominal pressure status postplication, angle of bed flexion before closure, and length of postoperative immobility all can contribute to increased venous stasis; all patients had rectus plication; and the OR table was flexed ∼30° at closure.^18^ To address the concern of additional expenses to the patient, all mechanical and chemoprophylaxis measures are provided at no extra cost to the patient. Preoperative heparin is provided by the hospital or the patient’s insurance. The home IPCs and access to the TouchMD service are provided by the operating surgeon.

## CONCLUSIONS

The results of our study suggest that the administration of comprehensive preoperative and postoperative mechanical and chemoprophylaxis is a safe and effective approach for mitigating mortality risk until a more sensitive risk stratification tool is available. Although the presented mechanical and chemoprophylaxis regimen proved effective in our cohort, further controlled, multicenter studies must be conducted to validate or invalidate the results.

## Supplemental Material

This article contains supplemental material located online at https://doi.org/10.1093/asjof/ojaf024.

## Supplementary Material

ojaf024_Supplementary_Data
