# Supplementary material for: A Comprehensive Mechanical and Chemoprophylaxis Algorithm for Prevention of Venous Thromboembolism in Lipoabdominoplasty
Source: Aesthet Surg J Open Forum. 2025 Apr 16;7:ojaf024. doi: 10.1093/asjof/ojaf024 (PMC12257947; doi:10.1093/asjof/ojaf024)
Supplement: ojaf024_Supplementary_Data [file ojaf024_supplementary_data.zip › SupplementalTable1 (1).docx]

| Concomitant procedure rate, N (%)  Bilateral mastopexy  Belt lipectomy  Umbilical hernia repair  Breast augmentation  Liposuction of trunk  Neo umbilicus  Lipo graft to breast  Liposuction of the thighs  Ventral hernia repair  Lipo graft to buttock  Breast reduction  Liposuction of upper flank  Replacing breast implants  Liposuction of chest  Liposuction of arms  Skin lesion excision  Lipo graft to face  Necklift  Facelift  Brachioplasty  Blepharoplasty  Fibroid removal  Gynecomastia  Liposuction of neck  Breast implant removal  Lumpectomy  Lateral trunkplasty | 223 (67.0%)  60 (18.0%)  44 (13.2%)  41 (12.3%)  33 (9.9%)  27 (8.1%)  19 (5.7%)  14 (4.2%)  12 (3.6%)  11 (3.3%)  11 (3.3%)  9 (2.7%)  6 (1.8%)  4 (1.2%)  4 (1.2%)  3 (0.9%)  3 (0.9%)  2 (0.6%)  2 (0.6%)  2 (0.6%)  2 (0.6%)  2 (0.6%)  1 (0.3%)  1 (0.3%)  1 (0.3%)  1 (0.3%)  1 (0.3%)  1 (0.3%) |
| --- | --- |

Supplemental Table 1. *Concomitant procedures performed at the time of abdominoplasty.*
